# Elemental Analysis of Coffee with Ion Beam Analytical Techniques

**DOI:** 10.3390/foods14040585

**Published:** 2025-02-10

**Authors:** Rafaela Debastiani, Leonardo Pessoa da Silva, Gabriela Corati Touguinha, Carla Eliete Iochims dos Santos, Livio Amaral, Johnny Ferraz Dias

**Affiliations:** 1 Institute of Nanotechnology, Karlsruhe Institute of Technology, Kaiserstr. 12, 76131 Karlsruhe, Germany; rafaela.debastiani@kit.edu; 2 Ion Implantation Laboratory, Institute of Physics, Federal University of Rio Grande do Sul, Av. Bento Gonçalves 9500, P.O. Box 15051, Porto Alegre CEP 91501-970, RS, Brazil; leonardo.pessoa@ufrgs.br (L.P.d.S.); gabrielactouguinha@gmail.com (G.C.T.); amaral@if.ufrgs.br (L.A.); 3 Graduate Program on Materials Science, Institute of Chemistry, Federal University of Rio Grande do Sul, Av. Bento Gonçalves 9500, Porto Alegre CEP 91501-970, RS, Brazil; 4 Physics, Statistics and Mathematics Institute, Federal University of Rio Grande, Rua Barão do Cahy 125, Santo Antonio da Patrulha CEP 9500-000, RS, Brazil; carlaiochims@yahoo.com.br

**Keywords:** ion beam analytical techniques, particle-induced X-ray emission, Rutherford backscattering spectrometry, coffee, coffee beans, roasted ground coffee, drip brewing

## Abstract

In this review, we present a compilation of results from studies of coffee carried out with accelerator-based analytical techniques employing swift ions. The fundamentals of these techniques are presented in detail. Moreover, different aspects of coffee are discussed, including the analysis of ground and roasted coffee beans, the effects of the drip brewing process on the final beverage, the importance of the water temperature for the extraction of elements during coffee preparation and how chemical markers can help discriminate coffee for forensic purposes. According to the experimental results, a matrix of different coffee types is represented by large amounts of carbon followed by mild amounts of oxygen. Moreover, elemental maps of roasted coffee beans show how the elements are distributed over the scanned area, thus providing valuable information on the co-localization of different elements within the beans. Concerning the drip brewing process, the results suggest that chlorine, potassium and phosphorus are quite soluble in hot water and therefore make their way into the drinking coffee. Moreover, the extraction of elements during the drip brewing process is dependent on the water temperature. The results obtained with ion-based techniques are discussed in perspective with those obtained by other analytical methods, including inductively coupled plasma technique in its various configurations. Advantages and drawbacks of these techniques are discussed. In this way, the present review opens up new possibilities for the analysis of coffee that go beyond traditional analytical techniques.

## 1. Introduction

Coffee is one of the most widely consumed beverages globally, largely due to its distinctive aroma and flavor and to the presence of caffeine, a natural stimulant present in coffee. The preparation of coffee involves roasting and grinding coffee beans, followed by infusion in warm water, filtration and percolation or pressing. Coffee is a complex compound that contains a variety of nutrients, including protein, lipids, carbohydrates, polyphenols, vitamins and minerals. These minerals include potassium, sodium, calcium, phosphorus, sulfur, chlorine, manganese, iron and zinc among others. In 2022, the world’s coffee production was about 10.8 billion kg [1] against 6.3 billion kg of tea [2]. It is estimated that more than two billion cups of coffee are consumed worldwide annually. Brazil is the world’s foremost producer and exporter of this agricultural commodity. Between 2023 and 2024, Brazil exported 41.4 million 60 kg bags of all coffee types, followed by Vietnam (28.6 million 60 kg bags), Colombia (10.9 million 60 kg bags) and India (6.5 million 60 kg bags) [3].

Ion beam analysis (IBA) [4] represents a group of techniques using swift ion beams for elemental analysis. These techniques are categorized into two distinct branches depending on the primary ion–matter interaction. Interactions involving the nuclei of the incident ion and of the target atom are classed as nuclear methods, such as RBS (Rutherford backscattering spectrometry) [5], ERDA (elastic recoil detection analysis) and NRA (nuclear reaction analysis) [6]. Conversely, interactions involving electronic excitations are termed atomic methods, such as PIXE (particle-induced X-ray emission) [7]. All these techniques are typically used in fields such as material science, biotechnology, medicine, physics and geology among others.

In the past 20 years, the combination of PIXE and RBS has been used for different purposes, including for the analysis of foodstuff in general. These studies include wine [[8] and references therein], tuna fish, table cream and tomato paste [[9] and references therein], fruits [8,9], vegetables [10], coffee [11,12,13] and grains [14,15,16], among others [17,18]. As PIXE and RBS require solid dry samples, liquid and pasty foodstuff have to go through a heat treatment prior to measurements, as described elsewhere [17]. These studies have demonstrated the versatility of the PIXE technique since one can characterize both solids and liquids samples with one single technique. In this way, PIXE can provide elemental concentrations throughout the production [17] and preparation [19] processes of foodstuff in general. Studies of coffee carried out with ion beam analytical techniques are summarized in Table 1.

The simultaneous combination of IBA techniques, usually referred as “total IBA” [24], can be employed to obtain the matrix, major, minor and trace elements of a sample. While RBS determines the matrix of a sample, which may consist of light elements that are usually not detected by conventional PIXE, the latter is sensitive to major and trace elements with concentrations in the range of mg·kg^−1^ [25]. The combination of these techniques bears great analytical advantage since both techniques are non-destructive. Moreover, the multi-elemental capability of PIXE and the good energy resolution of spectrometers enable fast access to information on the composition of a sample without previous knowledge of it. 

Even though IBA techniques are well established, they can be considered under the widespread umbrella of emerging technologies for specific niches. For instance, PIXE can provide unprecedented power of analysis for cases where different materials are part of any given study, e.g., on wine. With this technique, we can proceed with the elemental analysis of soil, roots, leaves, branches, grapes, wine, bottle glass and corks, thus covering the elemental characterization of the whole winemaking cycle with just one single analytical technique [8] and references therein]. Another niche is forensic science [12], since ion beam analytical techniques can provide non-destructive analyses, which is of utmost importance for cases brought to tribunals [26]. Finally, micro-PIXE excels at providing elemental maps of organic tissues due to its relatively low background when compared to scanning electron microscopy (SEM) images [13]. The spatial resolution achieved by the micro-PIXE technique is important for, e.g., studies of the translocation and internalization of chemical substances (elements, nanoparticles and molecules) in the cellular compartments of plants, grains and seeds and in any organic tissue [27,28]. In summary, IBA techniques can provide multi-elemental analysis of quite different materials in a non-destructive manner with good limits of detection and, finally, offer element localization capability within a sample through elemental maps.

The aim of the present review is to highlight the potentialities of ion-based techniques for the elemental analysis of coffee. In particular, it is shown that these techniques go beyond elemental analysis since they are non-destructive and have spatial resolution capability, thus filling the gap left by other techniques such as inductively coupled plasma (ICP), neutron activation analysis (NAA) and flame atomic absorption spectroscopy (FAAS). Aspects such as the elemental analysis of coffee beans and ground coffee and the discrimination of coffees from different brands and geographical locations are discussed. Moreover, the impact of different parameters on the extraction of elements from coffee are brought to scrutiny. Finally, a brief discussion of how PIXE results compare with those obtained by other techniques bring to light the advantages and disadvantages of them.

## 2. Analytical Techniques

### 2.1. Ion-Based Techniques

**Figure 1:**
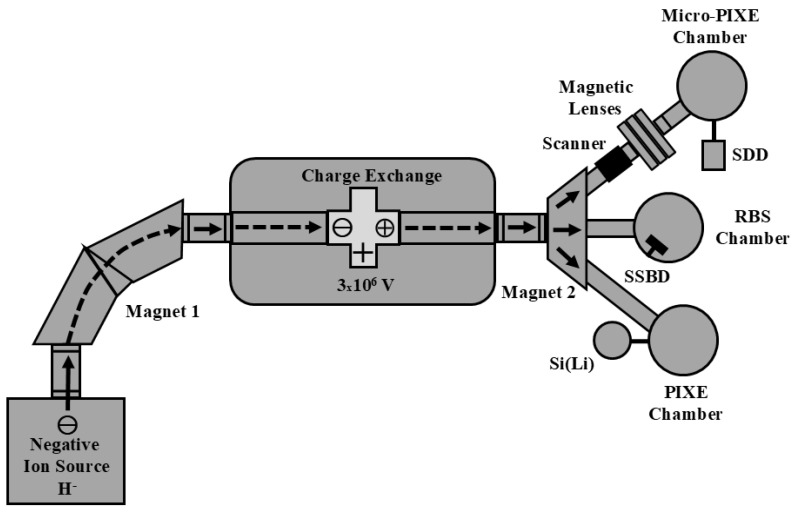
A schematic view of the Tandetron electrostatic accelerator at the Ion Implantation Laboratory used for PIXE, micro-PIXE and RBS experiments. See the text for further details. SDD: silicon drift detector; SSBD: silicon surface barrier detector; Si(Li): lithium-doped silicon detector. The arrows represent possible ion paths. All components are not to scale.

Techniques like PIXE, micro-PIXE and RBS rely on the use of swift ions, i.e., ions with energy in the order of a few million electron-volts (MeV). Therefore, an ion accelerator is needed to provide energetic ions. Although different types of accelerators can be used to achieve this goal, electrostatic accelerators like those from NEC (National Electrostatic Corporation) [29] and HVEE (High Voltage Engineering Europa) [30] are the ones of choice for analytical purposes. Figure 1 shows a simplified scheme of the 3 mega-volt (MV) Tandetron accelerator from HVEE installed at the Ion Implantation Laboratory (Federal University of Rio Grande do Sul). For a typical PIXE experiment, H^-^ ions are produced in the ion source and accelerated towards magnet 1 for the proper selection of the desired mass and charge. Subsequently, the negative ions penetrate the acceleration tube under an attractive force due to the positive terminal kept at a high potential (maximum of 3 × 10^6^ V) located at the center of the accelerator. Inside the charge exchange unit, the H^-^ ion loses its electrons, thus becoming positive H^+^. Now, the repulsion force between the positive terminal and the positive ion provides extra acceleration to the ion up to the desired energy. Finally, the positive ion exits the acceleration section and is directed to the PIXE beamline through the action of magnet 2. The acceleration procedure is similar for all ion-based techniques. Other techniques like RBS and micro-PIXE can be selected by magnet 2 as well. In the present setup, all experiments are conducted under vacuum. Typically, the pressure inside the PIXE, micro-PIXE and RBS chamber is of the order of 10^−6^ mbar.

PIXE is based on the detection of the characteristic X-rays emitted by the atoms of a sample when they are irradiated with energetic ion beams, usually protons of about 2–3 MeV [7]. Similarly, micro-PIXE is an analytical technique based on the same physical principles of conventional PIXE but makes use of a scanning system coupled to magnetic lenses (Figure 1) in order to provide elemental maps of the sample in the micrometer range or less [31,32]. In short, the MeV H^+^ ion beam from the accelerator interacts with the sample’s atoms, resulting in the ionization of the target atoms through the emission of inner-shell electrons to the continuum. The excited atoms can decay through the emission of characteristic X-rays representing different transitions between the electronic energy levels. The emitted X-rays are detected by energy-dispersive detectors and the corresponding electronic signals are processed in order to generate X-ray histograms as a function of the X-ray energy, usually given in units of kiloelectron-volts (keV). For PIXE and micro-PIXE experiments, a silicon detector doped with lithium (Si(Li)) or a silicon drift detector (SDD) is routinely used for the detection of X-rays. See Figure 1 for the experimental setup of the PIXE and micro-PIXE techniques.

**Figure 2:**
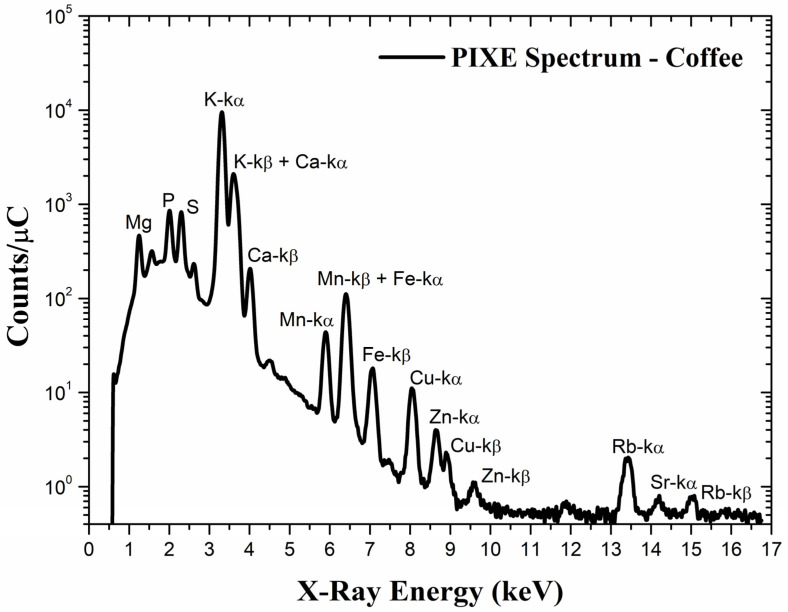
A typical PIXE spectrum for roasted ground coffee obtained with 2.0 MeV H^+^ ions. The counts are normalized by the charge accumulated during the experiment. X-ray transitions (k-α and k-β) are shown for elements heavier than sulfur. Overlaps of transitions are indicated as well.

Figure 2 depicts a typical PIXE spectrum of roasted ground coffee, showing several elements present in the sample. The number of X-rays related to a particular transition of an element (X-ray peak) can be converted to the element’s concentration in the sample with suitable software like GupixWin (version 3) [33]. In order to obtain absolute concentrations, several important physical parameters such as the ionization cross-section, detector’s properties, stopping power and matrix effects must be taken into account [7,33]. The knowledge of the matrix composition of each material analyzed by PIXE is crucial since matrix-related effects like X-ray self-absorption in the target and secondary fluorescence must be accounted for in calculations [7].

**Figure 3:**
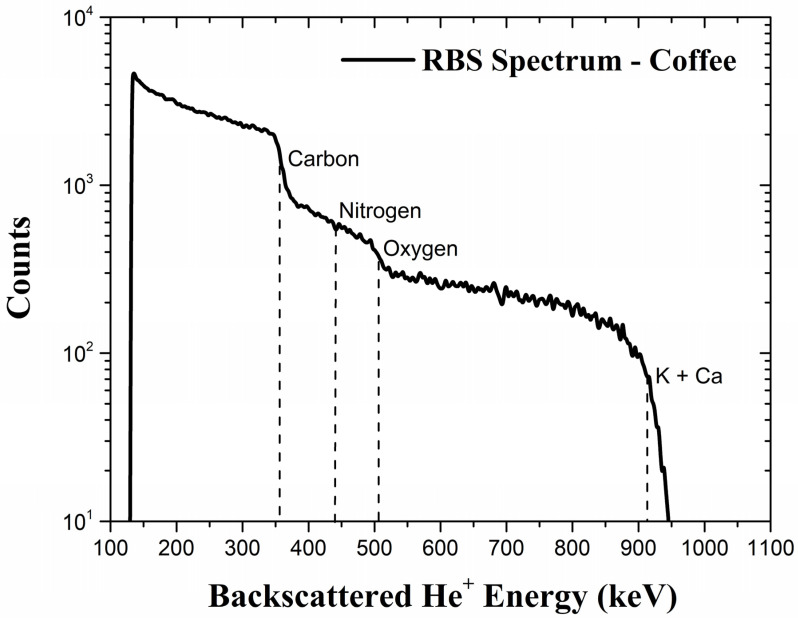
A typical RBS spectrum for roasted ground coffee obtained with 1.2 MeV He^+^ ions. Dashed lines indicate the edges corresponding to different elements present in the sample.

The RBS technique [5] can be used to determine the matrix of a sample under study. RBS is based on the backscattering of incident ions (usually He^+^ or He^++^) by an atom’s nuclei in a sample. During the backscattering event, the recoiling target nucleus keeps a fraction of the total energy of the incident particle. Therefore, the backscattered particle emerges from the target with lower energy due to the backscattering event and to the energy lost in the inward and outward paths inside the target. The backscattered ions are usually detected by silicon surface barrier detectors (SSBDs) (Figure 1). RBS is particularly suitable for the detection of light elements like C, N and O that cannot be quantified by PIXE. RBS spectra can be simulated by specific software like SIMNRA (version 7.03) [34] or PowerMEIS (version 3) [35,36] that takes into account important physical parameters and concepts such as the stopping power, Rutherford cross-sections, energy loss and energy straggling [5]. Figure 3 shows a typical RBS spectrum for roasted ground coffee, depicting the edges referring to C, N and O.

PIXE and RBS employ millimeter-size spot beams in order to probe the elemental concentrations of the sample under analysis. If the sample is homogeneous, the quantitative results are representative of the sample as a whole. However, it is also possible to obtain detailed information with spatial resolution in the micrometer range using a micro-PIXE setup (Figure 1). In this case, beams of a few square micrometers can produce elemental maps with their precise location in the material under analysis.

In general, the complexity of sample preparation for PIXE, micro-PIXE and RBS experiments depends on the materials to be analyzed and the technique to be used. Solid samples like paper filters used in the preparation of coffee through the drip brewing process can be placed in a special target holder for irradiation with no manipulation at all. Powder samples like roasted ground coffee and spent coffee must be homogenized and pressed into pellets about 2 mm thick. Since most experiments are carried out under low pressure (typically of the order of 10^−6^ mbar) inside the reaction chamber, liquid samples must go through a slow thermal procedure in order to dry out the sample. The remains of the sample are then homogenized and pressed into pellets as well. The details of this procedure can be found elsewhere [17].

### 2.2. Neutron Activation Analysis (NAA)

**Figure 4:**
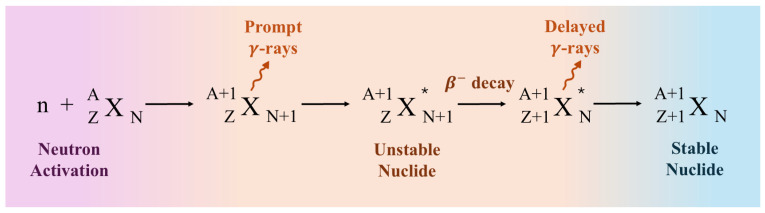
A neutron capture reaction featuring the emission of prompt and delayed gamma rays. See the text for further information.

Neutron activation analysis (NAA) [37,38] is an analytical technique based on the irradiation of a target material with neutrons in the non-elastic collision regime. The purpose of this technique is to produce unstable radioactive isotopes, which are formed through neutron capture reactions. Figure 4 illustrates the principle of the technique. Once a neutron is captured by the nucleus of an element, there could be the emission of prompt gamma rays, leaving behind an unstable nuclide. Since the unstable nuclide is in an excited state, it could decay through gamma emission and/or beta decay. Elemental quantification is obtained through the emission rate of gamma rays which is directly proportional to the concentration of the element. The identification of the element is determined by the measurement of the half-life of the nuclide and by the energies of the emitted gamma rays that are specific to each formed nuclide. Essentially, NAA is a multi-elemental technique that allows the simultaneous analysis of up to 70 elements with very high sensitivity for most of the elements. Moreover, it can be performed with very small amounts of material, down to just a few micrograms.

Research reactors with neutron fluxes of about 10^12^ neutrons per square centimeter per second are routinely used in NAA experiments. The detection system typically consists of high-resolution semiconductor detectors, and the experimental procedure can be optimized according to the type of sample being measured. This optimization takes into account the energy of the neutrons used in the reaction and the nuclide’s half-life, which affects the production and decay rate of gamma rays. Typically, NAA falls into two categories depending on what kind of gammas are detected. Prompt gamma neutron activation analysis (PGNAA) relies on the detection of prompt gammas during the irradiation. On the other hand, if delayed gammas are detected after the irradiation, then the technique is termed delayed gamma neutron activation analysis (DGNAA). In short, both PGNAA and DGNAA complement each other, as their applications depend on the nuclides produced by the neutron capture reaction. DGNAA is highly sensitive for the determination of most elements. However, it is not applicable for detecting gamma rays emitted by short-lived radionuclides, in which case PGNAA is ideal.

### 2.3. Inductively Coupled Plasma (ICP)

Inductively coupled plasma (ICP) [39,40,41] is a well-established method for the elemental analysis of materials which has found applications in several fields including biology and material science, among others. When combined with other analytical techniques such as optical emission spectrometry (OES), mass spectrometry (MS) and atomic emission spectrometry (AES), ICP becomes one of the most powerful analytical techniques.

The principle behind this technique involves subjecting a sample to high temperatures within a plasma region where, through atomization, excitation and ionization, the elemental concentration of the analyte can be determined. The plasma is generated within an inert gas flow, typically of argon, in a region submitted to a high-frequency (in the megahertz range) variable magnetic field induced by an alternate current passing through a coil. The ionization of argon occurs almost exclusively as Ar^+^, and the acceleration of these ions and electrons within this medium creates a highly efficient environment for atomizing the analyte. The sample is introduced either in a dissolved form or as a gas and is nebulized into the plasma region, where the analyte’s atoms go through excitation and de-excitation processes followed by the emission of electromagnetic radiation with a characteristic wavelength.

**Figure 5:**
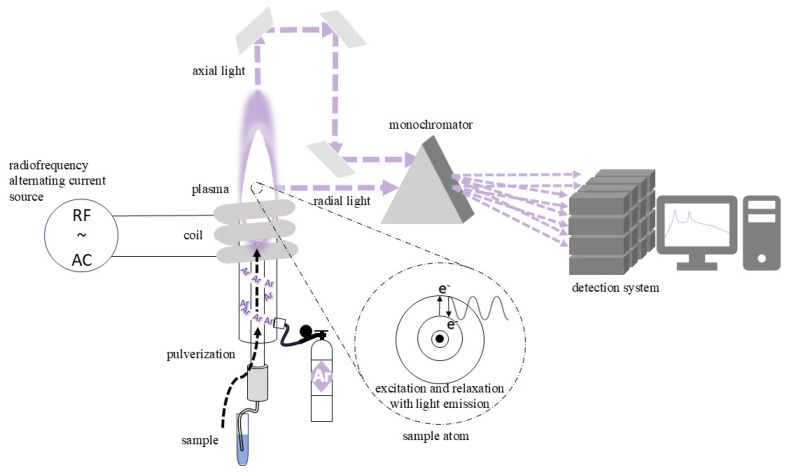
A schematic view of the main components of an ICP system. See the text for further information.

For the ICP-OES variant, the ionized and excited atoms in the high-temperature plasma region (around 10^4^ K) emit characteristic optical light as they return to lower energy states. The emitted light carries information about the concentration of the elements present in the analyte. Figure 5 shows a schematic view of a typical ICP-OES system. Radiation detection can be performed radially (lateral), axially or even dually (both radial and axial), each offering significant advantages and disadvantages in terms of sensitivity and matrix-induced interference. The detectable wavelength range typically varies from 200 to 600 nm, which covers more than 70 elements. For this purpose, monochromators with high-resolution capabilities are required. Once dispersed, the light is directed toward detectors such as photomultiplier tubes.

With proper calibration using standard samples and calibration curves, ICP-OES can identify trace elements in the ppm or ppb range. Moreover, it is multi-elemental since it can detect several elements simultaneously. Its main limitations include the destruction of the sample, spectral interference and relatively high operational costs. Promising future advancements in this technique involve the enhancement of plasma generation methods, such as microwave-induced plasma (MIP), which offer significant advantages over conventional ICP, such as reduced operating costs, increased sensitivity and minimized continuous background emission.

### 2.4. Atomic Absorption Spectroscopy (AAS)

**Figure 6:**
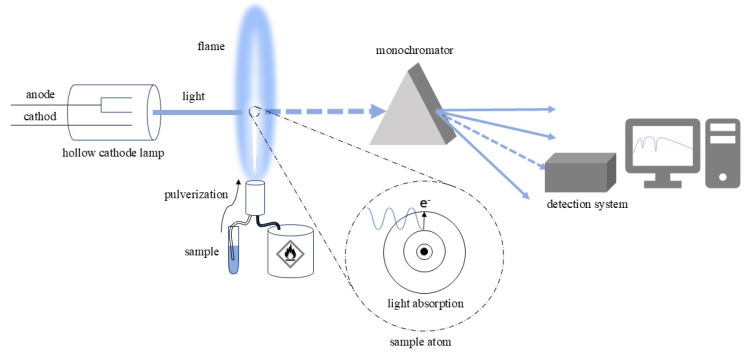
A schematic view of the main components of an FAAS system. See the text for further information.

Atomic absorption spectroscopy (AAS) [42] is a widely used analytical technique for the qualitative and quantitative determination of elements, especially metals and metalloids, in various types of samples. Figure 6 illustrates the technique, which is based on the absorption of radiation by an atomized sample. According to the Beer–Lambert Law, there is a relationship between the intensity of the absorbed radiation and the concentration of atoms in the light path.

The hollow cathode lamp is the most common radiation source and consists of a sealed tube filled with an inert gas (usually neon or argon), where the cathode is made of the same material to be analyzed (analyte). When an electric voltage is applied, the gas is ionized and the ions are accelerated toward the cathode, exciting the material’s atoms upon collision. The excited atoms emit characteristic radiation with a particular wavelength as it returns to its ground state, covering a discrete spectrum in the ultraviolet and visible ranges.

Sample atomization can be performed in various ways, with the most common method known as flame atomic absorption spectroscopy (FAAS), where the sample is pulverized into an aerosol and atomized in a flame generated by an acetylene burner. The flame’s properties must be adjusted to optimize atomization. However, there are variants of the atomization process, such as electrothermal atomic absorption spectroscopy (ETAAS), in which a high electric current applied to a graphite furnace significantly increases the temperature, thus promoting sample atomization.

The radiation with a characteristic wavelength generated by the lamp passes through the atomization region, where the analyte atoms are excited by the flame’s high temperature. Part of this radiation is absorbed by the excited atoms of the atomized analyte, leading to the de-excitation of the atoms. In this way, the absorption process decreases the intensity of the radiation from the lamp. The radiation is then directed by a lens to a monochromator, which selects the desired wavelengths and directs them to a detection system. Finally, the difference in radiation intensity measured with and without the atomized analyte is proportional to the concentration of the analyte.

FAAS is a valuable technique for analyzing a wide variety of samples, including geological, biological and food samples, among others. Its sensitivity is of the order of parts per million (ppm) when the atomization is performed using a flame and can reach up to parts per billion (ppb) when electrothermal atomization is applied. The technique has some limitations such as the need for calibration with standards, the destruction of the analyzed sample and the analytical restriction to one or a few elements at a time.

## 3. Results and Discussion

### 3.1. Ion-Based Techniques

#### 3.1.1. Coffee Matrix

The elemental concentrations obtained by PIXE must be corrected for matrix-related effects [7]. Coffee is an organic compound and, therefore, its matrix basically consists of light elements like carbon, nitrogen and oxygen.

**Figure 7:**
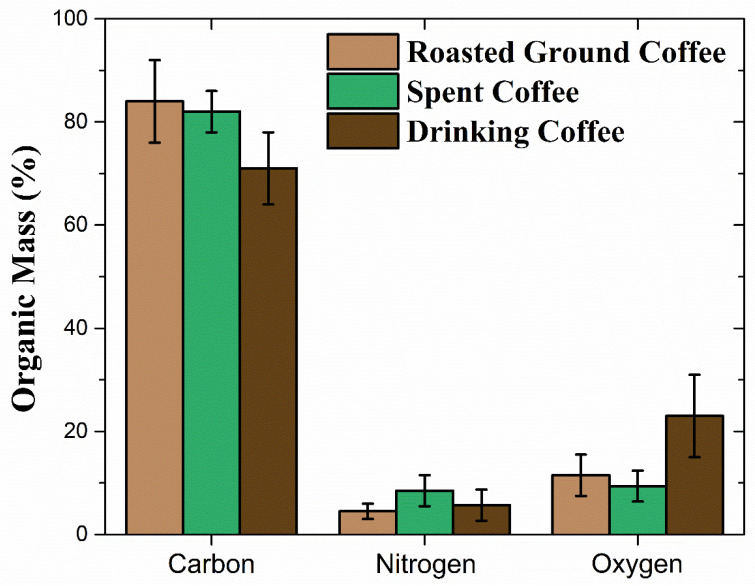
Matrix composition of roasted ground, spent and drinking coffees. Results are normalized for unity. Adapted from [13,19].

The matrix was evaluated for three different variants of coffee: (i) roasted ground coffee; (ii) spent coffee; (iii) and drinking coffee. These results are shown in Figure 7. The RBS results reveal that the roasted ground coffee consisted of ~84% C, ~4% N and ~12% O. Moreover, the largest variations in the matrices were observed for the drinking coffee, which featured a relatively smaller amount of C and a larger amount of O when compared to the other types of coffee.

#### 3.1.2. Coffee Beans

Green and roasted coffee beans from different geographical origins were analyzed using PIXE by Debastiani et al. [13], Cloete et al. [11] and Singh [20]. Cloete et al. [11] investigated the elemental composition, physical traits and the structural characterization of roasted coffee beans from Mexico, Ethiopia, Colombia and Honduras. Samples of roasted organic coffee beans were prepared by cross-sectioning the beans to explore their morphology without the use of any chemical preparation. For each of the coffee origins, 13 replicates were prepared and analyzed using an external beam PIXE setup, which did not require the use of a reaction chamber. The scanned areas were of the order of cm^2^.

**Figure 8:**
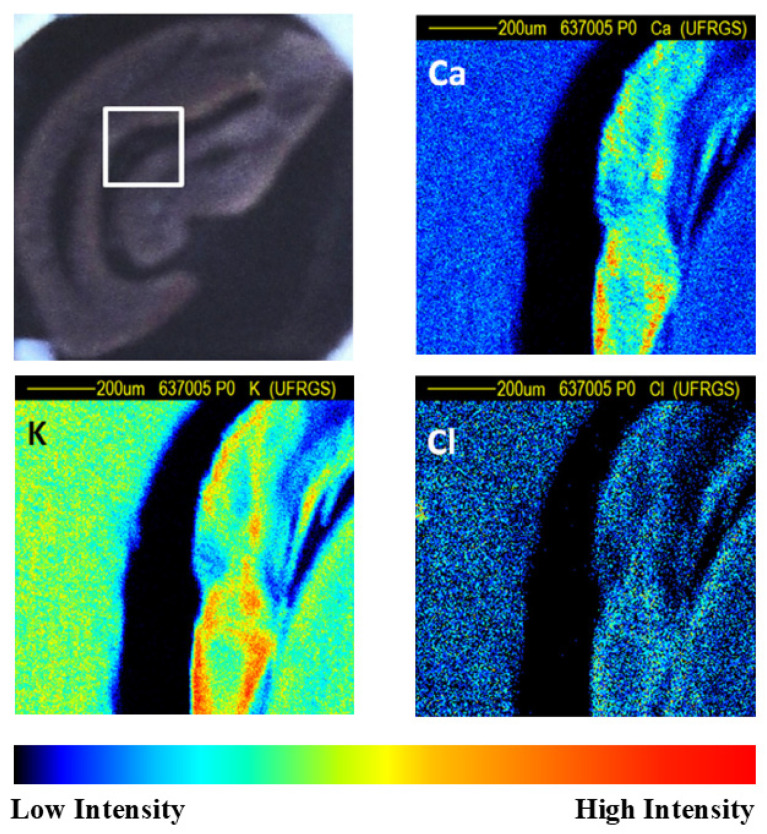
Elemental maps of Cl, K and Ca obtained with micro-PIXE from a sectional area of a whole roasted coffee bean. The proton energy was 2.2 MeV. The scanned area was 1 × 1 mm^2^. The top panel on the left shows a micrograph of the bean, while the white box depicts the scanned area. Adapted from [13].

Debastiani et al. [13] analyzed green and roasted coffee beans from Brazil using PIXE and micro-PIXE. For the analysis of roasted ground beans, 20 samples were prepared by grinding the beans and pressing the coffee powder into pellets. Whole green and roasted beans were analyzed with PIXE as well. In this case, six beans of each type were analyzed. Finally, sectional areas of the beans were cut for micro-PIXE analysis. In this case, the scan sizes varied from 500 × 500 µm^2^ up to 2000 × 2000 µm^2^ depending on the sample’s structure. Figure 8 shows some elemental maps from a sectional area of a whole roasted bean [13]. In this case, micro-PIXE revealed how elements like Cl, K and Ca were distributed across the bean. Hotspots with relatively high concentrations of K and Ca are visible in some regions of the bean, confirming the co-localization of these elements in the analyzed structure. This is a clear example of how micro-PIXE can help the understanding of the translocation and internalization processes of chemical substances in, e.g., plants [27,28].

**Figure 9:**
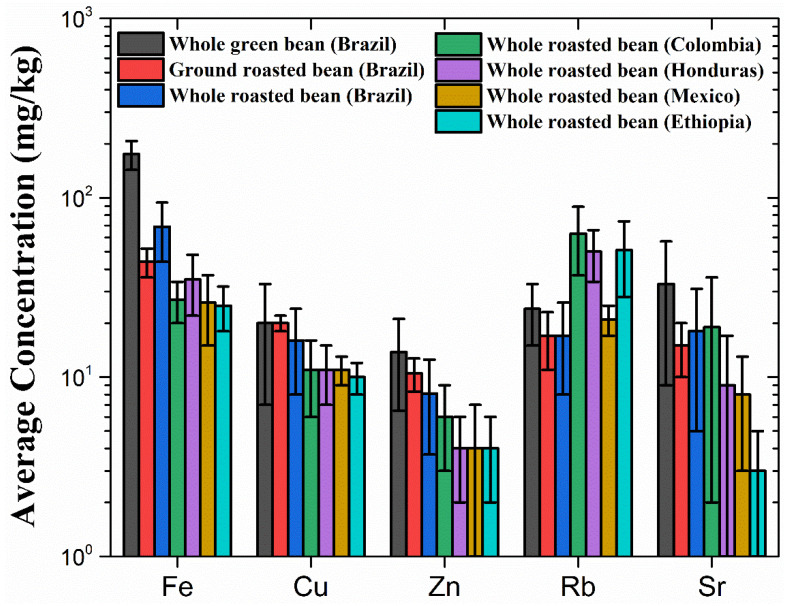
Concentrations of Fe, Cu, Zn, Rb and Sr in coffee beans from Brazil, Colombia, Honduras, Mexico and Ethiopia. The results are represented by the mean and the respective standard deviation. Values are given in mg·kg^−1^. Adapted from [11,13].

PIXE analysis detected P, S, Cl, K, Ca, Ti, Mn, Fe, Cu, Zn, Rb and Sr in coffee samples from Brazil [13], Colombia, Ethiopia, Mexico and Honduras [11]. In addition, Mg, Al and Si were detected in Brazilian coffee beans [13], while Br was found in some coffee beans analyzed by Cloete et al. [11]. The elemental maps indicated that K and Fe were not homogeneously distributed in the whole coffee beans [11,13]. Chlorine was homogeneously distributed, while potassium and calcium were more concentrated close to the edges of the structures for both green and roasted beans [13]. The level of inhomogeneities could be observed by the large variability in the results for the whole bean compared to the ground beans (see Table 2 and Table 3 from [13] and Table 2 and Figure 9 from this review).

The elemental composition of coffee beans can be used to determine particular geological features where the beans are grown. For instance, the Colombian coffee presented slightly higher concentrations of Rb and Sr compared to the coffees from the other four countries (Figure 9). These elements are known to play an important role in the geographic determination of coffee [43]. Interestingly, the concentration of Rb in the coffees from Colombia, Ethiopia and Honduras was three to four times higher than that found in the Brazilian coffee. Brazilian soil is known to have a high concentration of Rb, which is reflected in food produced there, including coffee and wine [17,22]. While the Colombian and Brazilian beans had the highest concentration of Sr, the Ethiopian beans had the lowest. The concentration of K was slightly larger for the ground roasted coffee beans from Brazil, while it was in the same range for the beans from the other countries. A large variation in the elemental concentration was observed for chlorine (Table 2), with the highest concentration found in the Mexican beans, which turned out to be around four times higher than the concentration found in the Brazilian beans. The concentration of Fe, Cu and Zn seemed to be larger for the Brazilian coffee, while it was similar in the coffee beans across Colombia, Honduras, Mexico and Ethiopia (Figure 9).

Green and roasted coffee beans were studied by Singh et al. [20] (Table 3). In this case, three species of coffee were selected for the study: *Coffea canephora* (Robusta coffee), *Coffea arabica* (Arabica coffee) and Peaberry coffee. For the PIXE analysis, bean samples were finely crushed and pelletized. In general, the lowest concentrations for most of the elements were observed in the roasted beans for all three species of coffee. Potassium and Ca were the elements with the highest concentrations for all coffee samples, while Cr, Ti, Mn, Fe, Ni, Cu, Zn, Rb, Sr and Se were found in trace amounts. No large differences were observed in the concentrations of trace elements between green and roasted beans or among coffee species. However, for major elements such as K and Ca, large variation was observed among the types and species of coffee. The concentration of K decreased from green to roasted beans for the Peaberry and Arabica species, while its concentration increased slightly for the Robusta coffee. The green beans of Peaberry coffee had the lowest concentration of K (22,540 mg·kg^−1^), while the Arabica coffee beans had the highest (29,967 mg·kg^−1^). Calcium had the lowest concentration for the Robusta coffee (2804 mg·kg^−1^). Phosphorus had twice the concentration in the green Arabica and Peaberry coffee in comparison to the green Robusta coffee, but for the roasted beans, the Robusta coffee showed the highest concentration among the three species. Moreover, Cl had about three times the concentration in the green Arabica coffee when compared to the Robusta and Peaberry coffees. The Fe concentration was much higher in the roasted Robusta coffee beans when compared to the other species and types, reaching a concentration of about 800 mg·kg^−1^ against 100–150 mg·kg^−1^ obtained for the other coffees. Overall, the Fe concentrations seemed to be much larger than the values found by Debastiani et al. [13] and Cloete et al. [11] (see Table 3 and Figure 9 for details). Moreover, the concentrations found for K were larger than the values reported in [11,13]. Calcium was found in the same range as obtained in [11,13], while P and Cl presented lower concentrations than the values reported in these studies. Finally, the concentrations of Rb found by Debastiani et al. [13] compared with those obtained by Singh et al. [20] (around 20 mg·kg^−1^), while Cloete et al. [11] observed Rb concentrations about twice as high (see Table 3 and Figure 9).

#### 3.1.3. Roasted Ground Coffee

Although coffee beans offer a higher-quality beverage when they are freshly ground right before the preparation of the coffee, they are more expensive and not as practical as ground and instant coffee options, which are the most popular choices for this beverage. This section will focus on studies using roasted ground coffee from different brands and batches of popular and gourmet coffees. Popular coffees usually do not have their region of production or the species of coffee described on the labels and are usually composed of coffees from different farms. On the other hand, gourmet coffees are sold as a specialty since they indicate the geographic region of production and are usually composed of the most tasteful Arabica beans. Most of the roasted ground coffee in this review corresponds to coffees produced in Brazil [12,13,19,21,22] and Jamaica [12] and three species from unknown origins [20].

PIXE analysis of different Brazilian brands [19] and different batches [21] of popular Brazilian roasted ground coffees indicated large differences in their elemental composition, suggesting that the influence of factors such as soil, environmental conditions, field practices and industrial processing conditions are important as far as elemental composition is concerned. These results demonstrate the complexity of determining the provenance for such products.

**Figure 10:**
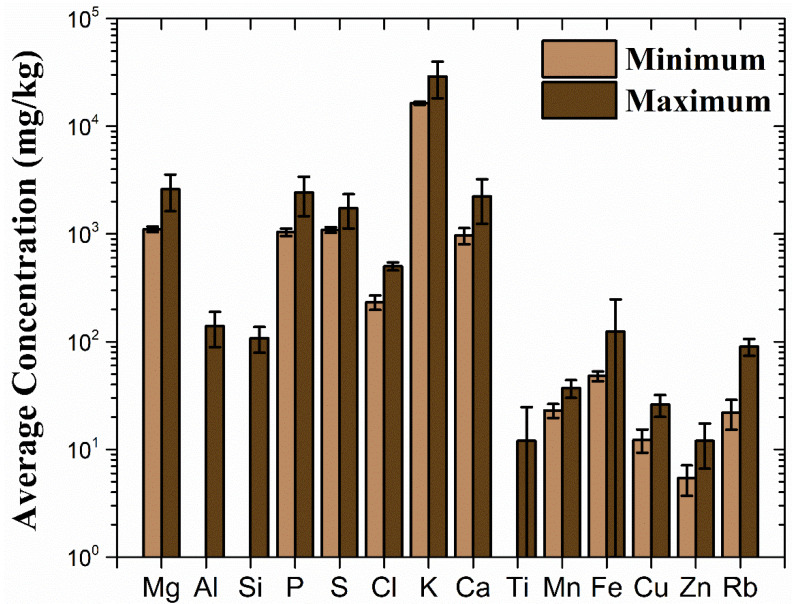
Minimum and maximum elemental concentrations found in popular Brazilian roasted ground coffees. Adapted from [12,13,19,21,22].

The study of eight brands of popular Brazilian roasted ground coffee was carried out using 3 packages from 2 distinct batches for each brand [19]. The results show large statistical differences across the brands for all the elements but Al, Si and Ti [19]. The lowest and the highest elemental concentrations were found in the Melitta and No Bule brands, respectively. For example, the concentrations of K and P varied from 18,142 mg·kg^−1^ and 1408 mg·kg^−1^ for the Melitta brand up to 29,039 mg·kg^−1^ and 2431 mg·kg^−1^ for the No Bule brand, respectively. The No Bule brand also showed large standard deviations. Interestingly, one of the No Bule packages showed concentrations from two to three times larger than the other two packages [19]. Such differences among batches led to a second study based on the analysis of eleven batches of Melitta Tradicional ground coffee produced over 2 years and 5 months [21]. Titanium was the only element to have a statistically equal concentration among the batches, while the remaining elements presented differences between at least two batches. For K and P, the concentrations varied from 17,821 mg·kg^−1^ and 1329 mg·kg^−1^ up to 22,994 mg·kg^−1^ and 1580 mg·kg^−1^ respectively, while the differences for the minor and trace elements like Cl, Ca, Ti, Mn, Fe, Cu, Zn and Rb among the batches were of about a factor of 2. Despite some exceptions, it was possible to sort out two distinct groups of batches, where one of them, corresponding to the first five batches, seemed to have relatively lower elemental concentrations than the other group, which corresponded to batches 6–11. These differences in batches of the same product indicate that the analysis employed for the determination of the origin of coffee requires advanced statistical tools, taking into account several parameters that impact elemental concentrations. The maximum and minimum mean elemental concentrations from popular Brazilian ground coffees are displayed in Figure 10. For Al, Si and Ti, the lowest concentration was below the limit of detection for some studies [12,13,19,21,22].

Coffees produced in particular regions of Brazil and Jamaica were analyzed in order to elucidate differences in their elemental compositions that might be correlated with their origin [10]. For the Brazilian coffee, three gourmet Arabica coffees with certified regions of provenance (Cerrado, Mogiana and Sul de Minas) and one popular Arabica–Robusta blended coffee (Melitta Tradicional) were analyzed using PIXE. While Mg, Si, P, S, Cl, K, CA, Mn, Fe, Cu, Zn and Rb were detected in most of the samples, Al, Sc, Ti, Co, Ni, Br and Sr were detected above the limit of detection (LOD) only in less than 50% of the samples. The frequency of the detection of the elements among coffee types could help in identifying potential discriminators of these coffees. For example, Sc and Co were absent in Melitta Tradicional, while Co was detected only in the Cerrado coffee. Therefore, these elements could constitute a potential chemical marker to distinguish these coffees from others. In addition, the concentrations of all elements but Al, Mg, Ni, Cu, Sr and Br were found to be different on statistical grounds from at least one of the coffees. Sulfur concentrations differed for all coffees, thus indicating that this element could also be used as a potential chemical marker for discrimination. Finally, coffees produced in geographically close regions, namely Mogiana and Sul de Minas, had statistically equal concentrations for Mg, P, K, Cl, Ca, Fe, Cu, Zn and Rb. Although Rb has been suggested to be a potential chemical marker for Brazilian organic products [22], it was detected above the LOD in only 42% of the Cerrado samples and in 89% of the Melitta Tradicional samples. Chlorine, which has been associated with moldy odor and musty taste in coffee [44], was found at lower concentrations for the gourmet coffees when compared to the Melitta Tradicional coffee. Such differences might be associated with the extra care taken for gourmet coffees during harvesting, processing and storage [12].

Regarding Jamaican coffees, three brands of the famous Blue Mountain coffees (Jablum, Island Blue and JBMC) were analyzed as well. Scandium, Co and Br concentrations were found to be below the LOD for all the Jamaican samples. The concentrations of Al, Si, Ti, Mn, Fe, Ni, Cu and Sr were similar for the three brands, but the occurrence frequencies of Al and Sr were less than 20% for at least one of the coffees. The concentrations of Rb were lower in comparison to the Brazilian coffees, while the Rb concentration found in the Island Blue coffee was below the LOD.

In order to compare the Brazilian and Jamaican coffees, the elemental concentrations of Melitta Tradicional and the average of all Blue Mountain concentrations were evaluated. Most of the elemental concentrations were statistically different between the coffees. Only Al, Si, Ni and Cu had similar concentrations. The concentrations of Mg, P, Cl, K, Mn, Fe and Rb were higher for the Brazilian coffee. Concerning Rb, its frequency of detection was just 18% for the Blue Mountain coffees and was much higher for Melitta Tradicional (89%).

Singh et al. [20] analyzed Arabica, Robusta and Peaberry ground coffee. Most of the elements seemed to have similar mean concentrations among the species, despite differences up to two times were observed for Sc, Mn, Rb and Se. From these elements, only Mn and Rb had all concentration values above the LOD. The highest concentrations of these elements were found in the Robusta coffee. Finally, the concentrations of Sc and Se are above the LOD only for the Arabica and Robusta coffees, respectively.

In summary, these studies pointed out similarities among coffees produced in neighboring regions, such as for Mogiana and Sul de Minas, and for the Blue Mountain coffees. Neighboring regions may share, to some extent, the same geology and climate, which can contribute to high degrees of similarity. On the other hand, these studies also highlighted differences observed in the elemental concentrations of coffees produced in different regions and countries. Furthermore, PIXE proved to be an excellent tool for finding potential chemical markers for the determination provenance, such as Rb, Sc and Co. These chemical markers, when combined with statistical methods and new machine learning tools, could be used for forensic purposes to identify the adulteration of coffee from a determined region of origin, thus protecting producers and consumers.

#### 3.1.4. Spent and Drinking Coffee

Spent and drinking coffee were studied through the drip brewing process in order to determine the elements transferred from the ground coffee to the beverage. The drip brewing process was chosen due to its popularity not only in Brazil but all over the world. The water temperature was 90 °C during this process. Pristine ground coffee and paper filters used during the preparation of the beverage were analyzed as well. Therefore, each stage of the process could be analyzed in terms of elemental composition [19] with one single technique, namely PIXE. As usual, RBS was also employed as an ancillary technique in order to determine the matrices of the materials used in the experiments.

Concerning the paper filter used during the preparation [19], its matrix was made of carbon (82%), nitrogen (1%) and oxygen (17%). Trace elements like Mg, Cl, Ca and Fe showed up with concentrations of the order of hundreds of parts per million. Finally, the results reported in [19] indicated that there was no significant transfer of elements from the filter to the beverage during the drip brewing process.

The analysis of ground, spent and drinking coffee groups consisting of 24 samples each revealed that elements such as Mg, P, K and Ca were the ones with highest concentrations in these groups, while Ti, Cu and Zn were the elements with the lowest concentrations (Table 4). The extraction factor (*EF*), i.e., the relative changes in the elemental concentration of spent coffee (*C_sc_*) with respect to that of ground coffee (*C_gc_*), is simply calculated as

$$EF=\frac{C_{gc}-C_{sc}}{C_{gc}}.$$

**Figure 11:**
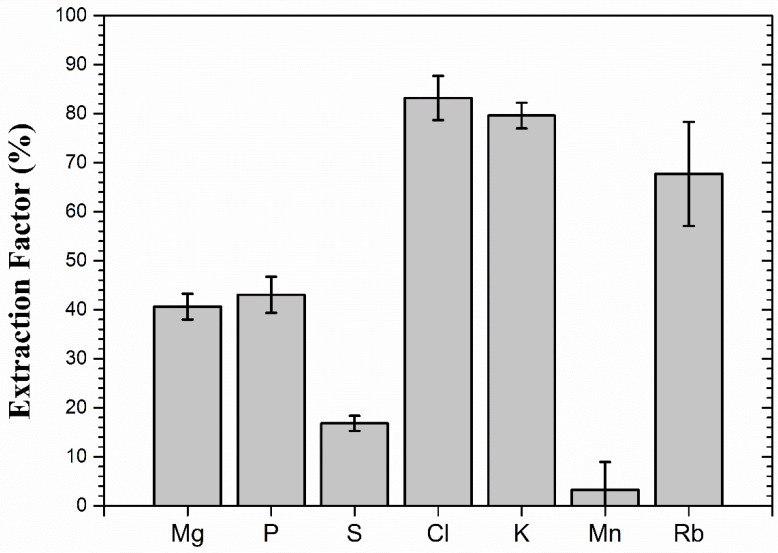
Extraction factors of some elements from coffee during the drip brewing process. See the text for further details. Adapted from [19].

Therefore, the extraction factor indicates the amount of elements extracted from the ground coffee and, in principle, transferred to the beverage. Figure 11 shows the positive extraction factors of some elements. Clearly, Cl, K and Rb were the elements with the highest extraction factors. However, it must be pointed out that, for some elements, the extraction factor may not have corresponded directly to transfer rates to the beverage. This could have been the case for Cl due to its high volatility.

Interestingly, there was also an apparent increase of about 15% in the concentrations of some elements, such as Ca, Ti, Cu and Zn, and of about 40% in the Fe concentration [19] in the spent coffee with respect to the ground coffee (Table 4). Although the concentrations of these elements were the same between the ground and spent coffees from a statistical point of view, these results suggest that these elements were not fully dissolved by the hot water and remained in the spent coffee. Since other elements were washed out from the spent coffee, their relative concentrations could have slightly increased. Moreover, it cannot be ruled out that the coffee absorbed elements from the water during its preparation since it may have carried elements from the components of the brewer.

A second study analyzed the extraction factor of elements from ground coffee as a function of water temperature [23]. Drip-brewed coffee was prepared with water temperatures varying from 20 °C to 80 °C, and samples of ground and spent coffee were analyzed with PIXE. The extraction factors obtained for several temperatures were compared to the results obtained at 90 °C [19]. Potassium, Rb and Cl were the elements with extraction factors of over 70% for all the temperatures studied. Phosphorus also presented large extraction factors for temperatures up to 45 °C, while decreasing factors were observed for higher temperatures. Similar behavior was observed for K, with a linear increase in the extraction up to 45 °C followed by an oscillating behavior at higher temperatures. Chlorine and Rb had similar extraction patterns but different extraction factors. As observed for the 90 °C case, elements like Ca, Cu, Si and Ti had their concentrations increase in the spent coffee for all temperatures as a consequence of poor solubility and absorption from the water. Curiously, for a few elements, both absorption and extraction factors were dependent on the temperature. Manganese was extracted from the coffee at all temperatures but 60 °C and 70 °C, which was compatible with the results obtained by Stelmach and collaborators [45] that found the lowest concentration of Mn in coffee prepared at such temperatures. Other elements with similar extraction and absorption behavior were Al, Fe and Zn [23].

### 3.2. Other Techniques

Besides ion-based techniques, other analytical techniques such as inductively coupled plasma (ICP) in its various formats (OES—optical emission spectrometry; AES—atomic emission spectrometry; MS—mass spectrometry) [46,47,48,49], neutron activation analysis (NAA) [49,50] and flame atomic absorption spectroscopy (FAAS) [47] have been routinely used for the analysis of plant-based products [51] and of food in general [52], including coffee. The main features of IBA and the techniques mentioned above are shown in Table 5. Typically, FAAS and ICP have better LODs (in the order of parts per billion or better) when compared to PIXE and NAA. Among all techniques, NAA presents better specificity for analysis. On the other hand, NAA experiments may take hours to days depending on the element to be probed. ICP and FAAS techniques require a more complicated sample preparation process, which could lead to chemical contamination and the incomplete dissolution of the sample, while NAA and PIXE usually require little sample handling prior to experiments. Moreover, NAA requires a nuclear reactor and the careful handling of samples due to the radioactivity induced in them [53,54,55]. In terms of cost, PIXE and NAA make use of sophisticated facilities with high maintenance costs.

It is important to point out that comparisons between ICP results and those obtained with micro-PIXE [56] and with PIXE [57,58] show a high degree of compatibility among these techniques. In addition, the results indicate that there is a clear complementarity between NAA and PIXE. Indeed, while PIXE fails to quantify elements with atomic numbers of Z ≤ 10 [59], it provides better results for elements within 15 ≤ Z ≤ 26 [60].

Table 6 shows some elemental concentrations of soluble coffee obtained with ICP [61], FAAS [62,63] and NAA [49]. With some exceptions, the overall agreement is quite good among the techniques. For the particular study, the NAA technique provided fewer results for the selected elements of Table 6. Iron, which is one of the essential elements for organic life [64] reached concentrations up to 451 mg·kg^−1^ [61]. Although even higher values were observed for Robusta coffee (Table 3) [30], the majority of Fe concentrations for all kind of coffees was around 100 mg·kg^−1^ or less and was the least for Brazilian coffees [19]. The concentrations obtained for Mg and Ca are compatible among all techniques. Trace elements like Cu and Zn are compatible as far as the ICP and FAAS techniques are concerned.

A similar comparison among techniques for roasted ground coffees is presented in Table 7. In particular, these kinds of coffee were measured in three independent laboratories [62,65,66] with the FAAS technique. It can be observed that the agreement between the FAAS results from references [62,65] is quite good for the concentrations of Mg, K, Ca, Mn, Fe and Cu. On the other hand, discrepancies for the concentrations of K and Mn as provided by FAAS are evident when the work of Ozdestan [66] is taken into account. The PIXE results show a good degree of compatibility with all techniques. However, significant discrepancies are observed for Fe (ICP—reference [49]), K (FAAS—references [62,65,66]) and Rb (NAA—reference [49]). PIXE and NAA provide consistent results for the concentrations of Mg, K, Ca, Mn, Fe, Zn and Rb.

Finally, it is important to bear in mind that the results shown in Table 6 and Table 7 refer to coffees of quite different origins. From this perspective, the overall agreement among ICP, FAAS, NAA and PIXE can be considered quite good.

## 4. Concluding Remarks

In recent decades, IBA techniques have proved to be suitable for the analysis of foodstuff in general and coffee in particular. Techniques like RBS, PIXE and micro-PIXE have the potential to analyze solid and liquid samples from, e.g., different steps of the production chain from the soil up to the final product [17]. Another important point is the reduced risk of chemical contamination since most of the samples can be analyzed in their pristine conditions with minimal handling or a simple heating treatment [13,17]. Moreover, PIXE is a non-destructive and fast multi-elemental technique, with limits of detection in the range of parts per million, being sensitive enough for most applications. In addition, the possibility of obtaining elemental maps through micro-PIXE and micro-RBS can greatly improve the understanding of the distribution of elements across microstructures of the samples [13]. However, it must be pointed out that ion beam analytical techniques require a particle accelerator to generate high-energy ion beams for analytical purposes, thus limiting the number of users globally. In this case, analysts are encouraged to cooperate with such laboratories since more than 300 electrostatic IBA facilities are scattered all over the world [68].

Like other analytical techniques, such as ICP, NAA and FAAS, PIXE can provide elemental concentrations for a great variety of materials. PIXE can identify chemical markers which may be a lead to specificity and selectivity. This is easily performed in some instances, like a particular contaminant in a product, and can be further improved by building a consistent database where different parameters, like the crop year, soil and field practices, are considered. By combining such databases with advanced statistical tools like multivariate and principal component analyses [69], it is possible to correlate a particular chemical marker to a particular elemental profile that occurs only in that particular type of product. Currently, the use of machine learning protocols coupled with artificial intelligence engines may solve the quest for the absolute global differentiation of organic products including coffee. Finally, it has been shown elsewhere [12] that the combination of IBA techniques with others, like Fourier transform infrared (FTIR) and radiocarbon analysis with accelerator mass spectrometry (AMS-^14^C) techniques, becomes a powerful tool for selectivity.

In summary, the studies presented in this review show that IBA techniques can be used for the analysis of foodstuff and beverages in general. As far as coffee is concerned, analysis can be carried out in different stages of production (green and roasted beans, ground coffee) and of beverage preparation (spent and drinking coffee). Moreover, the elemental maps provided by micro-PIXE are particularly important for determining the precise location of elements in different parts of plants and beans. By combining IBA techniques with proper statistical tools, chemical markers can be identified and used for discrimination among brands and the geographical origins of products under study, thus illustrating the potential of these techniques to be used for forensic purposes.

## Figures and Tables

**Table 1:** A summary of data published on coffee obtained with ion beam analytical techniques. The “beverage” coffee type refers to drinking coffee [19], while “filter” refers to the paper filter after the preparation of the beverage with roasted ground coffee through the drip brewing process [20]. NA: information not available.

Coffee Type	Coffee Origin	Technique/Imaging	Quantitative Elemental Analysis	Reference
Green beans	Brazil	PIXE/No	Mg, Al, Si, P, S, Cl, K, Ca, Ti, Mn, Fe, Cu, Zn, Rb, Sr	[13]
Green beans	Brazil	Micro-PIXE/Yes	Cl, K, Ca, Fe	[13]
Roasted beans	Brazil	PIXE/No	Mg, Al, Si, P, S, Cl, K, Ca, Ti, Mn, Fe, Cu, Zn, Rb, Sr	[13]
Roasted beans	Brazil	Micro-PIXE/Yes	Cl, K, Ca, Fe	[13]
Roasted beans	Ethiopia Colombia Honduras Mexico	External beam PIXE/Yes	P, S, Cl, K, Ca, Ti, Mn, Fe, Cu, Zn, Br, Rb, Sr	[11]
Green beans	NA	PIXE/No	P, S, Cl, K, Ca, Sc, Cr, Ti, Mn, Fe, Cu, Zn, Rb, Sr, Se, Ni	[20]
Roasted beans	NA	PIXE/No	P, S, Cl, K, Ca, Sc, Cr, Ti, Mn, Fe, Cu, Zn, Rb, Sr, Se, Ni	[20]
Roasted ground	Brazil	RBS/No	C, O, N	[13,19]
Roasted ground	Brazil	PIXE/No	Mg, P, S, Cl, K, Ca, Mn, Fe, Cu, Zn, Rb, Sr	[13]
Roasted ground	Brazil	PIXE/No	Mg, Al, Si, P, S, Cl, K, Ca, Ti, Mn, Fe, Cu, Zn, Rb	[21]
Roasted ground	Brazil	PIXE/No	Mg, Al, Si, P, S, Cl, K, Ca, Ti, Mn, Fe, Cu, Zn, Rb	[22]
Roasted ground	Brazil	PIXE/No	Mg, Al, Si, P, S, Cl, K, Ca, Ti, Mn, Fe, Cu, Zn, Rb	[23]
Spent	Brazil	PIXE/No	Mg, Al, Si, P, S, Cl, K, Ca, Ti, Mn, Fe, Cu, Zn, Rb	[23]
Roasted ground	Brazil	PIXE/No	Mg, Al, Si, P, S, Cl, K, Ca, Ti, Mn, Fe, Cu, Zn, Rb	[19]
Spent	Brazil	RBS/No	C, N, O	[19]
Spent	Brazil	PIXE/No	Mg, Al, Si, P, S, Cl, K, Ca, Ti, Mn, Fe, Cu, Zn, Rb	[19]
Beverage	Brazil	RBS/No	C, N, O	[19]
Beverage	Brazil	PIXE/No	Mg, Al, Si, P, S, Cl, K, Ca, Ti, Mn, Fe, Cu, Zn, Rb	[19]
Filter	NA	PIXE/No	P, S, Cl, K, Ca, Sc, Cr, Ti, Mn, Fe, Cu, Zn, Rb, Sr, Se, Ni	[20]
Roasted ground	Brazil Jamaica	PIXE/No	Mg, Al, Si, P, S, Cl, K, Ca, Sc, Ti, Mn, Fe, Co, Ni, Cu, Zn, Br, Rb, Sr	[12]

**Table 2:** Some elemental concentrations of coffee beans from Brazil, Colombia, Honduras, Mexico and Ethiopia. The results are represented by the mean and the respective standard deviation. Values are given in mg·kg^−1^. Adapted from [11,13].

Element	Whole Green Bean (Brazil)	Ground Roasted Bean (Brazil)	Whole Roasted Bean (Brazil)	Whole Roasted Bean (Colombia)	Whole Roasted Bean (Honduras)	Whole Roasted Bean (Mexico)	Whole Roasted Bean (Ethiopia)
P	627 ± 94	1205 ± 121	934 ± 343	766 ± 521	977 ± 665	900 ± 319	995 ± 643
S	2021 ± 416	1209 ± 44	1660 ± 295	1328 ± 397	1934 ± 694	1724 ± 401	1936 ± 594
Cl	290 ± 245	173 ± 25	138 ± 61	309 ± 175	390 ± 270	662 ± 184	527 ± 111
K	14,011± 4048	19,104 ± 562	16,372 ± 4643	16,543 ± 3632	16,805 ± 4503	17,577 ± 2265	17,315 ± 2610
Ca	3366 ± 2768	1633 ± 401	2019 ± 1481	1686 ± 758	1371 ± 391	1574 ± 960	1205 ± 447
Mn	55 ± 51	41 ± 3	36 ± 27	32 ± 17	23 ± 9	21 ± 6	16 ± 6

**Table 3:** Some elemental concentrations of green and roasted beans of Peaberry, Robusta and Arabica coffees. Values are given in mg·kg^−1^. Adapted from [20].

Element	Green Peaberry	Roasted Peaberry	Green Robusta	Roasted Robusta	Green Arabica	Roasted Arabica
P	320 ± 32	150 ± 25	163 ± 26	329 ± 33	328 ± 30	222 ± 57
Cl	65 ± 5	56 ± 5	77 ± 4	90 ± 6	207 ± 6	77 ± 5.2
K	22,540 ± 24	16,056 ± 24	24,170 ± 24	25,463 ± 30	29,967 ± 27	19,548 ± 25
Ca	3167 ± 38	1152 ± 29	2804 ± 39	1599 ± 42	3732 ± 48	1452 ± 34
Fe	104 ± 1	105 ± 2	131 ± 2	819 ± 4	147 ± 1	108 ± 2
Rb	18 ± 3	12 ± 3	28 ± 3	28 ± 4	26 ± 3	16 ± 6

**Table 4:** Some elemental concentrations of ground, spend and drinking coffees. The results are represented by the mean and the respective standard deviation. Values are given in mg·kg^−1^. Adapted from [19].

Element	Ground Coffee	Spent Coffee	Drinking Coffee
Mg	1715 ± 133	1018 ± 166	97 ± 23
P	1437 ± 138	819 ± 212	77 ± 18
K	20,970 ± 1104	4278 ± 2294	1419 ± 297
Ca	1450 ± 187	1699 ± 364	38 ± 8
Ti	7.4 ± 2.4	8.3 ± 6.1	0.16 ± 0.06
Cu	19.6 ± 3.9	23 ± 4	0.13 ± 0.07
Zn	9.9 ± 2.8	11.9 ± 3.4	0.21 ± 0.05

**Table 5:** Main features of the PIXE, RBS, FAAS, ICP and NAA analytical techniques.

Technique [Ref.]	Sensitivity	Quantifiable Elements	Specificity	Analysis Time	Relative Cost	Advantages	Disadvantages
PIXE [7]	ppm	Na-U	High	A few minutes per sample	High	Multi-elemental analysis Non-destructive Complements other IBA techniques Minimal sample preparation (no need for chemical treatment) Allows the analysis of different materials from a study with a single analytical technique Imaging capability with high spatial resolution	High cost Limited access as it requires a particle accelerator Requires solid samples for in-vacuum experiments
RBS [5]	ppm	B-U	High	5–30 min per sample	High	Multi-elemental analysis Non-destructive Complements other IBA techniques Minimal sample preparation (no need for chemical treatment) Depth profiling capability for surface analysis Imaging capability with high spatial resolution	High cost Limited access as it requires a particle accelerator Limited sensitivity for trace elements Requires solid samples
FAAS [51,52]	ppb	Metals and a few metalloids	Moderate to high	A few minutes per element	Low	Affordable Easy access (many facilities around the world) High sensitivity	Single element analysis Sample preparation requires dilution and acid digestion Not suitable for non-metal analysis
ICP [51,52]	ppb-ppt	Over 70 elements can be detected	Moderate to high	Minutes per sample	Moderate to high	Multi-elemental analysis Isotope detection capability	Can require complex sample preparation Matrix and spectral interferences may lead to inaccuracies
NAA [51,52,55]	ppm	Over 70 elements can be detected	Very high	Hours to days per sample	High	Multi-elemental analysis Non-destructive Minimal sample preparation Matrix influence is often negligible Target non-homogeneity is negligible Bulk analysis capability	Requires access to a nuclear reactor or high-energy neutron source Post-irradiated samples require extra care due to potential radioactivity

**Table 6:** Some elemental concentrations of soluble coffee obtained with ICP, FAAS and NAA analytical techniques. Values are given in mg·kg^−1^. Some results are quoted as minimum and maximum values [49,61,63]. Some figures were rounded off for the sake of clarity. See the text for further information. NA: information not available. Adapted from [49,61,62,63].

Element	ICP [Ref.]	FAAS [Ref.]	NAA [Ref.]
Mg	2120–4150 [61]	3400 ± 1070 [62] 2000–3100 [63]	3207–3846 [49]
Al	NA	NA	18–22 [49]
P	2230–4100 [61]	4130 ± 855 [62]	NA
S	1480–2060 [61]	NA	NA
K	32,500–47,600 [61]	25,300 ± 5270 [62] 13,600–29,100 [63]	35,600–38,900 [49]
Ca	1060–1890 [61]	1160 ± 609 [62] 490–971 [63]	1352–2059 [49]
Mn	4–39 [61]	18 ± 8 [62] 7–13 [63]	NA
Fe	14–451 [61]	34 ± 15 [62] 16–92 [63]	NA
Cu	0.5–2.3 [61]	0.7 ± 0.3 [62] 0.4–16.0 [63]	NA
Zn	3–15 [61]	4.0 ± 3.5 [62] 2–9 [63]	NA

**Table 7:** Some elemental concentrations of roasted ground coffee obtained with ICP, FAAS, NAA and PIXE analytical techniques. Values are given in mg·kg^−1^. Some results are quoted as minimum and maximum values [67]. Some figures were rounded off for the sake of clarity. See the text for further information. NA: information not available. Adapted from [19,49,62,65,66,67].

Element	ICP [Ref.]	FAAS [Ref.]	NAA [Ref.]	PIXE [Ref.]
Na	NA	NA	56 ± 1 [49]	NA
Mg	NA	2100 ± 435 [62] 1964 ± 78 [65] 2097 ± 449 [66]	2133 ± 4 [49]	2092 ± 323 [19]
P	1394 ± 1 [49]	2280 ± 496 [62]	NA	1761 ± 303 [19]
Cl	NA	NA	NA	384 ± 79 [19]
K	NA	13,690 ± 1020 [62] 14,488 ± 467 [65] 9263 ± 1188 [66]	21,400 ± 3000 [49] 20,800–22,700 [67]	22,451 ± 3436 [19]
Ca	NA	841 ± 331 [62] 945 ± 65 [65]	1381 ± 5 [49] 1151–1408 [67]	1437 ± 303 [19]
Ti	NA	NA	NA	8 ± 3 [19]
Mn	NA	22 ± 6 [62] 23 ± 1 [65] 49 ± 6 [66]	29 ± 2 [49]	32 ± 8 [19]
Fe	32 ± 1 [49]	42 ± 14 [62] 52 ± 4 [65]	55–250 [67]	68 ± 23 [19]
Cu	19 ± 1 [49]	16 ± 2 [62] 14 ± 1 [65]	NA	18 ± 5 [19]
Zn	6 ± 1 [49]	5 ± 3 [62] 15 ± 1 [65]	6–8 [67]	9 ± 2 [19]
Rb	NA	NA	8 ± 4 [49] 37–57 [67]	48 ± 20 [19]

## Data Availability

No new data were created or analyzed in this study. Data sharing is not applicable to this article.
